# Tissue Distribution of Doxycycline in Animal Models of Tuberculosis

**DOI:** 10.1128/AAC.02479-19

**Published:** 2020-04-21

**Authors:** Martin Gengenbacher, Matthew D. Zimmerman, Jansy P. Sarathy, Firat Kaya, Han Wang, Marizel Mina, Claire Carter, Md Amir Hossen, Hongwei Su, Carolina Trujillo, Sabine Ehrt, Dirk Schnappinger, Véronique Dartois

**Affiliations:** a Center for Discovery and Innovation, Hackensack Meridian Health, Nutley, New Jersey, USA; b New Jersey Medical School, Rutgers, The State University of New Jersey, Newark, New Jersey, USA; c Weill Cornell Medical College, New York, New York, USA

**Keywords:** doxycycline, TetR, tuberculosis, animal models, tissue penetration, genetic regulation *in vivo*

## Abstract

Doxycycline, an FDA-approved tetracycline, is used in tuberculosis *in vivo* models for the temporal control of mycobacterial gene expression. In these models, animals are infected with recombinant Mycobacterium tuberculosis carrying genes of interest under transcriptional control of the doxycycline-responsive TetR-*tetO* unit. To minimize fluctuations of plasma levels, doxycycline is usually administered in the diet. However, tissue penetration studies to identify the minimum doxycycline content in food achieving complete repression of TetR-controlled genes in tuberculosis (TB)-infected organs and lesions have not been conducted. Here, we first determined the tetracycline concentrations required to achieve silencing of M. tuberculosis target genes *in vitro*. Next, we measured doxycycline concentrations in plasma, major organs, and lung lesions in TB-infected mice and rabbits and compared these values to silencing concentrations measured *in vitro*. We found that 2,000 ppm doxycycline supplemented in mouse and rabbit feed is sufficient to reach target concentrations in TB lesions. In rabbit chow, the calcium content had to be reduced 5-fold to minimize chelation of doxycycline and deliver adequate oral bioavailability. Clearance kinetics from major organs and lung lesions revealed that doxycycline levels fall below concentrations that repress *tet* promoters within 7 to 14 days after doxycycline is removed from the diet. In summary, we have shown that 2,000 ppm doxycycline supplemented in standard mouse diet and in low-calcium rabbit diet delivers concentrations adequate to achieve full repression of *tet* promoters in infected tissues of mice and rabbits.

## Abstract (precis)

Doxycycline, an FDA-approved tetracycline, is used in tuberculosis *in vivo* models for the temporal control of mycobacterial gene expression. In these models, animals are infected with recombinant Mycobacterium tuberculosis carrying genes of interest under transcriptional control of the doxycycline-responsive TetR-*tetO* unit. To minimize fluctuations of plasma levels, doxycycline is usually administered in the diet. However, tissue penetration studies to identify the minimum doxycycline content in food achieving complete repression of TetR-controlled genes in tuberculosis (TB)-infected organs and lesions have not been conducted.

## TEXT

Transcriptional regulators derived from the tetracycline repressor (TetR) of Escherichia coli have been exploited for more than 2 decades to chemically regulate gene expression with tetracyclines in eukaryotic cells (1–3). The specificity of the Tet repressor-operator-effector interaction and the favorable *in vivo* pharmacokinetics of tetracyclines make this regulatory system well suited for the temporal control of gene expression *in vivo*, such as in transgenic animals (4) and animals infected with recombinant pathogenic bacteria carrying genes of interest under the TetR-*tetO* transcriptional control unit (5–7).

Promoters that can be controlled by TetR and tetracyclines were initially developed *in vitro* in Gram-positive and Gram-negative bacteria (8). More recently, such systems have also been developed for medically relevant acid-fast bacteria, including M. tuberculosis (9, 10). Owing to its excellent oral bioavailability and pharmacokinetic properties in mice and its relative lack of activity against M. tuberculosis with an MIC of 8 to 16 μg/ml (11), doxycycline (DOX) has frequently been used in tuberculosis (TB) mouse models to induce or repress M. tuberculosis genes (12–15). In general, the affinity of DOX for TetR is several orders of magnitude higher than its affinity for the ribosome, providing a safe range within which genetic regulation can be achieved without affecting bacterial growth (16).

To regulate M. tuberculosis gene expression during infection, DOX is administered either in the drinking water at up to 2 mg/ml or in commercially available modified diets at 200 to 2,000 ppm, where it was reported to be stable for at least 7 days and 6 months, respectively (17, 18). Supplementation of DOX in mouse feed is considered the most convenient method to achieve adequate and consistent plasma levels (18).

Since DOX is commonly used as a broad-spectrum antibiotic in veterinary medicine and the food industry, tissue distribution studies have been conducted in animals such as horses and broiler chickens to assess its penetration into various organs and cell types. DOX shows favorable tissue partitioning and equilibration of free drug concentrations (with a free fraction in plasma of 20%) between plasma and interstitial fluid at steady state. Accumulation in specific cell types, such as polymorphonuclear leukocytes (19) and fibroblasts (20, 21), has been observed. However, no tissue penetration study has been conducted to determine whether adequate DOX levels are achieved in TB-infected organs and immune cell types in which the pathogen resides.

This work was undertaken to (i) identify the range of DOX concentrations that achieve full repression of Tet-OFF promoters *in vitro*, (ii) measure DOX concentrations in major organs and in lung lesions in TB-infected mice and rabbits in order to determine the lowest DOX feed content required to achieve the target concentrations identified *in vitro*, and (iii) explore the clearance kinetics of DOX from lung tissue and lesions to determine the number of days required for DOX levels to fall below concentrations sufficient to induce or repress *tet* promoters after DOX is removed from the diet.

## RESULTS

### Concentrations required for control of Tet-OFF constructs by tetracyclines *in vitro*.

**FIG 1:**
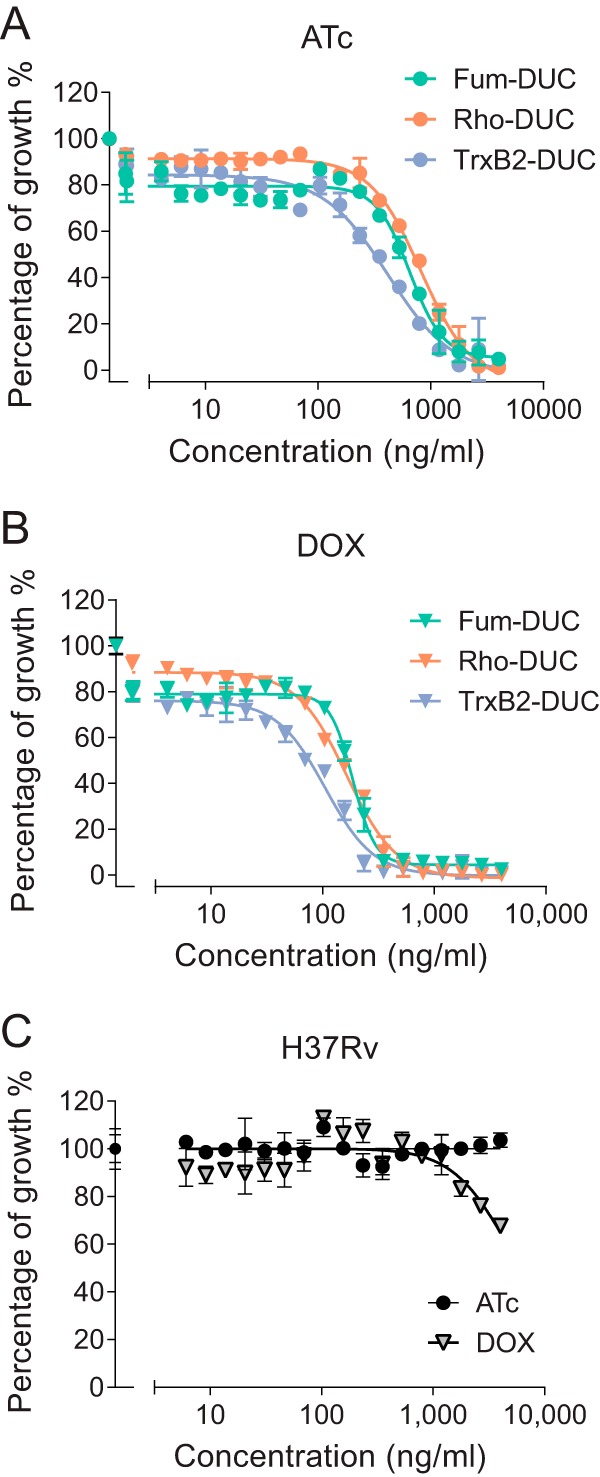
Effect of tetracyclines on *in vitro* growth of M. tuberculosis H37Rv harboring essential genes under TetR control compared to wild type. (A and B) Growth inhibitory dose response of anhydrotetracycline (ATc) and doxycycline (DOX) in M. tuberculosis strains that harbor a TetR-dependent dual-control genetic switch (14) driving expression of Fum (fumarate hydratase, green symbols), Rho (transcription termination factor, orange symbols), or TrxB2 (thioredoxin reductase, blue symbols). (C) Growth inhibitory dose response of ATc and DOX against wild-type M. tuberculosis H37Rv.

Anhydrotetracycline (ATc) is conventionally used as the *in vitro* inducer or repressor of TetR-*tetO* constructs due to its lack of growth inhibitory activity against M. tuberculosis. DOX, on the other hand, is the preferred transcriptional regulator *in vivo* because it is orally bioavailable and inhibits M. tuberculosis growth only at high concentrations (11). To determine the concentrations of ATc and DOX required to achieve complete silencing of target genes in M. tuberculosis *in vitro*, we resorted to genetically engineered strains in which a dual-control switch combining repression of transcription and controlled proteolysis had been chromosomally integrated to silence gene and protein activities (14, 22–24). Three M. tuberculosis strains, each with a dual-control switch targeting either *fum*, *rho*, or *trxB2* (three essential M. tuberculosis genes), were exposed to increasing concentrations of ATc or DOX. ATc concentrations ranging from 1 to 2 μg/ml were required to achieve 90% growth inhibition resulting from effective silencing in all three strains (Fig. 1A). When DOX was used as the TetR ligand *in vitro*, 50% growth inhibition was achieved at 80, 120, and 200 ng/ml for *trxB2*, *rho*, and *fum*, respectively; 90% growth inhibition was achieved between 230 and 350 ng/ml (Fig. 1B). Thus, lower DOX than ATc concentrations are required to control TetR-*tetO* constructs in M. tuberculosis. To ensure that these concentrations do not interfere with the growth of wild-type M. tuberculosis and to confirm published MICs, full dose-response curves were obtained with M. tuberculosis H37Rv exposed to both tetracyclines. M. tuberculosis growth remained unaffected by ATc up to the highest concentration tested (4 μg/ml), while DOX achieved approximately 30% growth inhibition at 4 μg/ml (Fig. 1C), consistent with the published MICs of 8 to 16 μg/ml (11). These results indicate that the concentrations required to achieve maximum control of TetR-*tetO* constructs do not affect growth of M. tuberculosis *in vitro*.

### Pharmacokinetics and tissue distribution of DOX in mice.

**FIG 2:**
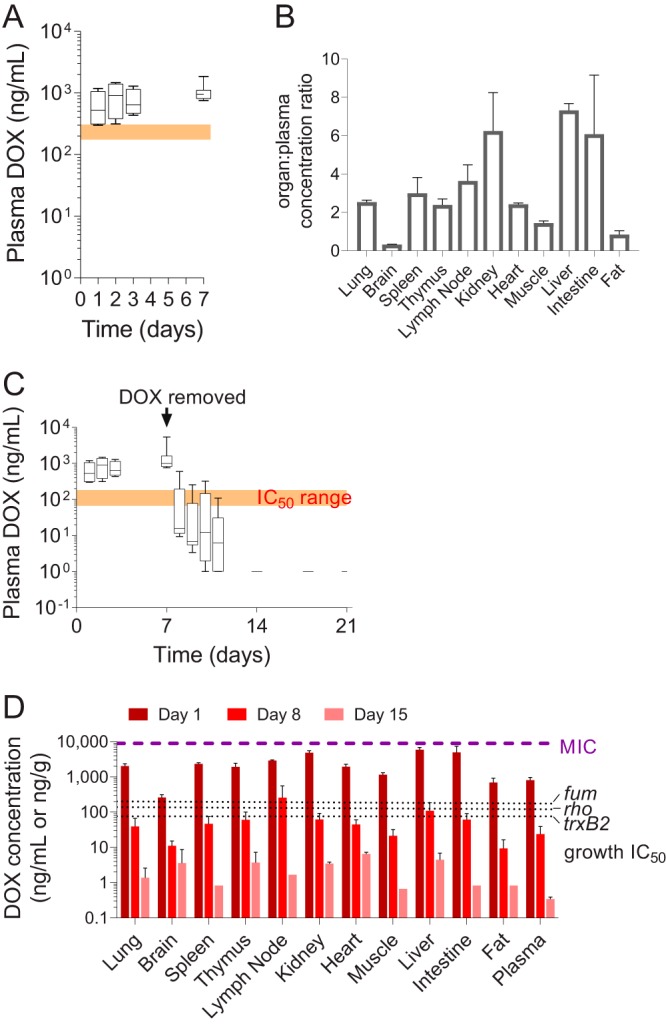
Plasma and tissue pharmacokinetics of DOX in mice receiving DOX-supplemented diet. (A) Box and whisker plot of DOX plasma concentrations in mice receiving 2,000 ppm DOX in standard diet for 7 days. Mean values (bar), minimum and maximum (box) and standard deviations (error bars) are shown for *n* = 4 to 11 mice per group. The orange shaded window indicates the range of *in vitro* DOX concentration that inhibits 90% of bacterial growth (IC_90_) for the three reporter strains shown in Fig. 1. (B) DOX concentrations in mouse organs relative to plasma after 7 days on the DOX diet. Mean values and standard deviations (error bars) are shown (*n* = 3). (C) Kinetics of DOX clearance in plasma following removal of DOX from the diet for 14 days. Mean values (bar) and minimum and maximum (box) and standard deviations (error bars) are shown (*n* = 3 to 12). The orange shaded window indicates the range of *in vitro* DOX concentrations that inhibit 50% of bacterial growth (IC_50_) for the three reporter strains shown in Fig. 1. (D) Kinetics of DOX clearance from major organs of TB-infected mice 7 and 14 days after removal of DOX from the diet. Day 1 corresponds to DOX levels in mice that had received a DOX-supplemented diet for 1 week, after which it was replaced by a DOX-free diet for the duration of the experiment. The purple dotted line indicates the lower boundary of published MIC ranges against wild-type M. tuberculosis (11), and the black dotted lines show individual DOX IC_50_s for the three reporter strains (Fig. 1). Mean values and standard deviations (error bars) are shown (*n* = 3).

To compare the DOX concentrations achieved in mice relative to DOX MICs and concentrations required to regulate TetR-controlled promoters, we first measured DOX concentrations in the plasma of TB-infected mice receiving 2,000 ppm DOX in the diet. Plasma was collected on days 1, 2, 3, and 7 following the switch from DOX-free to DOX-medicated chow, 3 weeks postinfection with M. tuberculosis strain H37Rv. Plasma levels of DOX ranged from 300 to 1,190 ng/ml (average, 637 ng/ml; standard deviation [SD], 411 ng/ml) on day 1 and reached an average of 1,081 ng/ml (SD, 386 ng/ml; range, 757 to 1,850 ng/ml) on day 7 (Fig. 2A), thus achieving the concentrations required to inhibit 90% of the growth of strains harboring representative Tet-OFF-controlled essential genes *in vitro* (Fig. 1B). To assess potential lot-to-lot variability in DOX chow, groups of 3 mice were fed three different lots, and plasma DOX concentrations were measured in serial blood samples from 0 to 5 h on day 1 (see Fig. S1 in the supplemental material), showing consistent plasma levels across lots. Actual DOX concentrations were measured in each lot and found to be within 25% of the target content of 2,000 ppm (Table S1).

To quantify penetration into tissues, we measured DOX concentrations in all major organs in the TB-infected mice that had received DOX-medicated chow for 7 days (Fig. 2B), showing favorable penetration in all organs except for brain tissue. In infected lung, spleen, and lymph node homogenates, total DOX concentrations were 2- to 4-fold higher than in plasma, thus exceeding levels required to achieve 90% growth inhibition, while not reaching the MIC of 8 to 16 μg/ml (Fig. 2B). To determine the time required for DOX to clear plasma and infected organs below concentrations sufficient to induce or repress Tet-ON and Tet-OFF promoters, mice were switched to a DOX-free diet after 7 days on DOX chow, and DOX concentrations in plasma were measured at 1, 2, 3, 4, 7, 11, and 14 days post-DOX removal (Fig. 2C). To determine how this translated within tissue, we quantified DOX in homogenized organs from 3 mice per group at 1, 8, and 15 days post-DOX removal, showing that concentrations fell below the levels required to achieve 50% growth inhibition in all organs except lymph nodes within 8 days post-DOX removal (Fig. 2D).

### Dose finding pharmacokinetics of DOX in rabbits.

**FIG 3:**
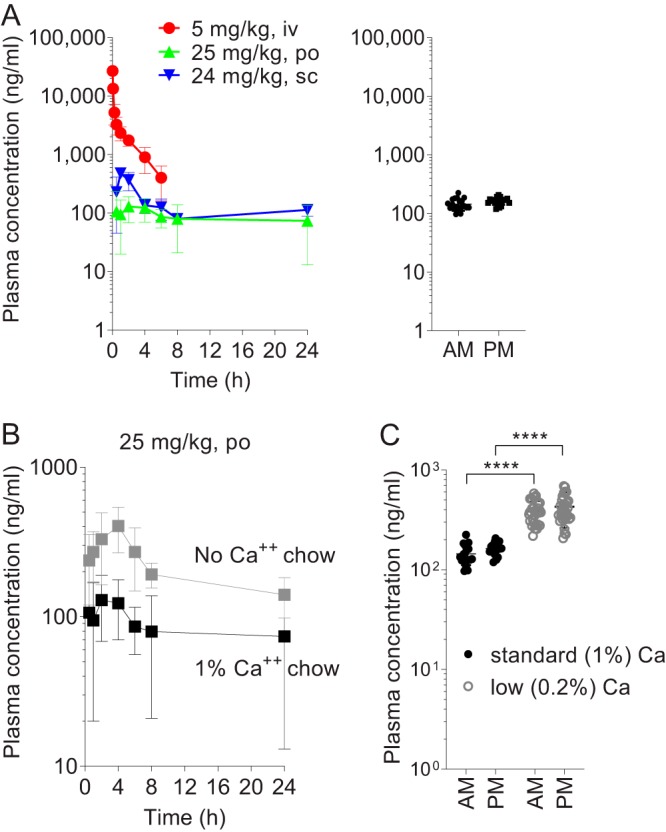
Plasma and tissue pharmacokinetics of DOX in rabbits receiving DOX-supplemented chow. (A) Left panel: DOX concentration-time profile in rabbit plasma following a single DOX dose administered intravenously (i.v.), by oral gavage (p.o.), and subcutaneously (s.c.), as indicated. Right panel: DOX plasma concentrations in rabbits receiving 2,000 ppm DOX in standard chow for 7 days. AM, immediately prior to receiving daily DOX-supplemented feed in the morning; PM, 6 h after receiving DOX feed. (B) Effect of dietary calcium removal on the plasma pharmacokinetics of DOX in rabbits following a single oral dose of 25 mg/kg. (C) Effect of dietary calcium lowering (0.2% total calcium content, or 5-fold lower than in standard chow) on DOX plasma levels in rabbits that received 2,000 ppm DOX in chow for 5 to 7 days. AM and PM time points are as described for right panel A. Data are represented as scatter dot plots of individual animal values and are pooled from two separate experiments with 4 and 6 rabbits, respectively (horizontal bar indicates mean, *n* = 20 to 34 blood samples from 10 rabbits).

The rabbit model of TB disease is well suited to assess the essentiality and vulnerability of bacterial targets in mature cellular and necrotic lesions resembling those found in human TB (25, 26). However, DOX pharmacokinetics (PK) and distribution in pulmonary lesions have not been characterized in rabbits. Preliminary PK studies in rabbits that received a modified diet with 200 and 400 ppm DOX showed unexpectedly low plasma levels (Fig. S2A). When the DOX content of the rabbit diet was increased to 2,000 ppm (corresponding approximately to 100 mg/kg), plasma levels increased proportionally, but remained lower than in mice, at 100 to 200 ng/ml throughout the 10-day PK study (Fig. 3A).

To determine whether poor oral bioavailability or species-specific metabolism was the reason for the low DOX concentration in rabbits, we dosed uninfected rabbits with DOX via the intravenous (i.v.), oral (p.o.), and subcutaneous (s.c.) routes. Following bolus i.v. dosing, a biphasic concentration-time profile was observed in plasma, with a distribution and elimination phase, consistent with a two-compartment PK model (Fig. 3A, Table 1). The volume of distribution at steady state (steady-state volume of distribution [*V*_dss_] or volume of distribution [*V*_d_] area) was high, reaching a maximum of approximately 1 liter/kg, corresponding to total body volume and similar to the *V*_d_ measured in domestic rabbits (27) and in pigs (28). The clearance was low at 26 ml/min or 15% of liver blood flow (Table 1). The i.v. concentration time profile and PK parameters were consistent with published data that used a bioassay for DOX quantitation following i.v. injection in domestic rabbits (27). DOX administration by oral gavage resulted in 3.2% bioavailability (Fig. 3A, Table 1), indicating that poor absorption rather than fast metabolism may partially account for the low oral bioavailability. To determine whether pharmaceutical grade formulation might improve oral absorption, we dosed rabbits with DOX tablets at approximately 15 and 30 mg/kg, which did not result in detectable exposure improvement (Fig. S2B). Lastly, subcutaneous injection resulted in a 1.5-fold increase in the area under the concentration-time curve (AUC) and average concentration throughout the dosing interval (Fig. 3A, Table 1).

**TABLE 1:** Pharmacokinetic parameters of DOX in rabbits^*a*^

Route	Dose (mg/kg)	AUC_0–24_ (ng/h/ml)	C_ave_ (ng/ml)	Elim T_1/2_ (h)	*k*_el_ (h^−1^) (liters/kg)	V_dss_/*V* area (liters/kg)^*b*^	CL (ml/min)	F (%)
Intravenous bolus	5	12,478 (4,058)	NA	1.95 (0.49)	0.35 (0.08)	1,056/1,138	26.2 (5.6)	NA
Oral gavage	25	2,046 (973)	85 (40)					3.2
Subcutaneous	24	3,175 (356)	132 (15)					5.2
Oral gavage (standard 1% calcium chow)	25	2,317 (275)	97 (11)					3.6
Oral gavage (calcium-free chow)	25	5,014 (950)	209 (40)					7.9
Oral gavage (TB-infected)	20	360	15					0.7

a AUC_0–24_, area under the concentration-time curve; Elim T_1/2_, elimination half-life; C_ave_, average plasma concentration over 24 h; *k*_el_, elimination rate constant; CL, clearance; F, bioavailability.

b V_dss_ = dose · [(A/alphâ2 + B/betâ2)/AUĈ2]; *V* area = dose/beta kel · AUC (45).

Calcium found in dairy products interferes with tetracycline oral absorption because it forms insoluble chelates resulting in decreased bioavailability (29). While most mammals tightly regulate blood calcium, rabbits absorb nearly all dietary calcium and excrete the excess through the kidneys. This results in a surge of blood calcium following food intake (30), proportional to calcium levels in the diet. We therefore hypothesized that DOX absorption and plasma concentrations may increase in rabbits receiving a calcium-free or low-calcium diet. Rabbits were fed a calcium-free or 1% calcium diet for a minimum of 2 days, at which point they received a single 25-mg/kg dose of DOX via oral gavage. As hypothesized, exposure (AUC) after a single dose was twice as high in rabbits on a calcium-free diet (Fig. 3B, Table 1).

To determine whether a similar improvement in DOX exposure would be seen when DOX is provided in the chow, under dietary conditions compatible with long-term experiments (i.e., not calcium-free), we placed rabbits on either standard (1% calcium) chow with DOX at 2,000 ppm or 0.2% calcium chow with DOX at 2,000 ppm, as described in Materials and Methods. Blood samples were collected twice a day (a.m. and p.m.) after 5 to 7 days on DOX chow as indicated. The respective mean a.m. and p.m. plasma DOX concentrations were 144 and 163 ng/ml in the standard-calcium group and 385 and 429 ng/ml in the low-calcium group (Fig. 3C), showing a 2 to 3-fold increase in the low-calcium group and reinforcing the trend observed when DOX was received as a single oral dose. Having optimized constant DOX exposure when provided in the diet, we next studied its distribution in the cellular and necrotic compartments of mature rabbit lesions in order to determine the DOX concentrations to which M. tuberculosis bacilli are exposed in tissues.

### *In vitro* lesion pharmacokinetics.

Previous work by our group indicated that drug distribution and partitioning within lesions is a function of uptake into macrophages and binding to caseum macromolecules (31, 32). To predict the penetration of DOX in cellular lesions and its behavior at the interface between the cellular rim and necrotic core of TB lesions, we measured DOX uptake into macrophages and caseum binding. DOX uptake was quantified in THP-1 macrophages, where it reached an intracellular to extracellular concentration ratio of 4.1 (SD, 0.6). This favorable cellular uptake predicted good penetration into cellular lesions. The average unbound drug fraction in caseum (*f*_u-caseum_) was moderate to high at 12 to 14% (SD 3%) of total drug. Together with the moderate uptake in macrophages, the DOX fraction unbound predicted favorable partitioning at the cellular/caseum interface and good passive diffusion through nonvascularized caseum. Taken together, these results suggest that DOX should reach higher concentrations in both cellular and necrotic lesions than plasma.

### Distribution of DOX in cellular and necrotic lesion compartments.

**FIG 4:**
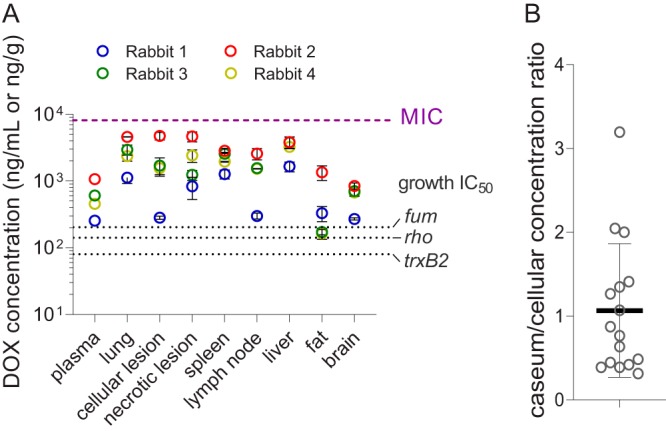
DOX penetration in major organs, lung lesions, and necrotic caseum of TB-infected rabbits. (A) DOX concentrations in plasma, uninvolved lung tissue, lung lesions, and major organs in 4 rabbits receiving a low-calcium 2,000 ppm DOX diet for 7 days. Mean values and standard deviations (error bars) are shown (*n* = 3). (B) Scatter dot plots of caseum-to-cellular DOX concentration ratios in necrotic granulomas collected from TB-infected lungs. Ratios obtained in 10 lesions from 5 infected rabbits are shown.

Uninvolved lung, cellular, and necrotic lesions were dissected from the lungs of TB-infected rabbits receiving 2,000 ppm DOX in low-calcium (20% of standard or 0.2% final) chow for 7 days. DOX was also quantified in spleen, lymph nodes, liver, fat, and brain tissue. We found higher DOX levels in cellular and necrotic lesions than in plasma (Fig. 4A), indicating that DOX accumulates in infected tissues relative to plasma, as predicted by the macrophage uptake assay and consistent with the reported accumulation in polymorphonuclear leukocytes (19). Favorable penetration was also measured in spleen, liver, and lymph nodes. All tissue concentrations were above the levels required to achieve 90% growth control, with the exception of one animal with borderline DOX concentrations in some of the tissues. These lower concentrations were explained by low food consumption (Table S2). DOX levels were below the M. tuberculosis MIC in all tissues (Fig. 4A). To quantify the partitioning of DOX at the cellular/caseum interface, we used laser-capture microdissection in thin sections of large necrotic lesions (33) from the same rabbits and found caseum-to-cellular DOX concentration ratios ranging from 0.3 to 3.2 with a mean value of around 1.0 (Fig. 4B). Overall, we found (i) higher DOX concentrations in fully cellular lesions than in plasma in line with the macrophage uptake data and (ii) comparable concentrations in caseum and cellular rims, confirming the favorable passive diffusion of DOX through avascular caseum predicted by the caseum binding assay. Collectively, these results indicate that in rabbits on low-calcium chow supplemented with DOX at 2,000 ppm, DOX concentrations are sufficient to achieve complete repression of Tet-OFF genetic constructs but not high enough to affect growth and viability of wild-type M. tuberculosis.

## DISCUSSION

We have shown that 2,000 ppm DOX supplemented in a standard mouse diet and in a low-calcium rabbit diet deliver DOX concentrations adequate to achieve full repression of representative Tet-OFF promoters in infected tissues of both mice and rabbits. These concentrations remain below the DOX MIC in all tissues and are thus likely to primarily affect M. tuberculosis growth and viability by regulation of genes under the control of TetR. The clearance kinetics of DOX from lung lesions and other infected organs indicate that DOX levels fall below concentrations sufficient to repress *tet* promoters within 7 days after DOX is removed from the diet.

DOX bioavailability is species specific and subject to food effect. It is very poor in rats (34) and moderate to low (20%) in pigs (28) but almost complete in calves (35) and humans (36). In nonfasted rabbits that received 150 mg/kg tetracycline by oral gavage (27), bioavailability was estimated at 4% (Data Set S1), in line with the present findings. Interestingly, DOX exposure was also very low in rabbits that received subcutaneous injections as part of this work, indicating that low bioavailability is not exclusively due to poor oral absorption. Tetracyclines are known to localize in bones and teeth, where 99% of the body calcium is found and where they form calcium-bound depots. They also accumulate in liver and spleen, where they are more highly bound than in plasma (http://www.cyto.purdue.edu/cdroms/cyto2/17/chmrx/tetra.htm). Such trapping in selected tissues could contribute to species-specific plasma levels. In early studies, the distribution of DOX in tissues was described in rats (37) and in patients undergoing biopsies during surgery (38). DOX concentrations were measured in plasma/serum and in major organs using a bioassay and indicated lung-to-serum concentration ratios between 2 and 3, consistent with our findings. Penetration was highest in kidneys and intestines and lowest in fat tissue (37, 38), again in line with our results (Fig. 2B).

Rabbits possess a unique calcium metabolism. Most mammals regulate blood calcium to maintain levels within a narrow range by controlling the absorption of calcium from the diet according to need. Rabbits have adopted the different strategy of absorbing nearly all dietary calcium ingested and renally excreting the excess. As a result, blood concentrations transiently reflect calcium levels in the diet, and calcium carbonate is excreted in large amounts in the urine (30). It has been determined that 0.2% calcium in rabbit chow is sufficient to sustain normal growth and weight gain (30), whereas standard diets contain 1% calcium. Reducing calcium content to 0.2% in rabbit chow supplemented with 2,000 ppm DOX led to improved oral bioavailability and at least 2-fold higher plasma levels throughout the day. DOX concentrations in all relevant organs, in lung lesions, and in plasma were higher than the minimum required to regulate *tet* promoters and did not reach the lower threshold of published MICs in broth (8 to 16 mg/liter [11]). Similar MICs have been measured against intracellular M. tuberculosis in macrophages, and bactericidal activity was observed at 40 mg/liter (39). DOX showed favorable partitioning into the necrotic foci of caseous lesions, with an average caseum/cellular concentration ratio of 1/1. Since caseum M. tuberculosis is nonreplicating and overall phenotypically resistant to most TB drugs, DOX is not expected to affect M. tuberculosis viability in this lesion compartment. Given the similar penetration of DOX in uninvolved lung, cellular, and caseous lesions (Fig. 4A), partial lesion resolution over time due to effective gene silencing by DOX and reduction of bacterial burden is unlikely to impact DOX penetration. Slow tissue clearance has been reported for DOX (40), consistent with our observation that levels fall below effective concentrations after approximately 7 days on a DOX-free diet. These findings have implications as to the kinetics of *tet* regulation following DOX removal and should be taken into consideration when interpreting related data.

Many tetracyclines, including DOX, have anti-inflammatory properties (41) mediated by suppression of tumor necrosis factor-α (TNF-α) and matrix metalloproteases (MMP). *In vitro*, DOX inhibits secretion of MMPs induced by TB infection at 5 mg/liter and higher (39). Thus, fine-tuning the DOX dose in both mouse and rabbit diets to reach but not exceed effective concentrations is important to minimize anti-inflammatory side effects and potential gastrointestinal disturbances.

## MATERIALS AND METHODS

### *In vitro* repression of Tet promoters.

The construction of the ATc/DOX-controlled M. tuberculosis mutants (Fum-DUC, Rho-DUC, and TrxB2-DUC) has been described (22–24). Mutant strains were grown to mid-log phase and diluted to an optical density (OD) of 0.03 in 7H9 medium. Bacteria were then exposed to 1.5-fold serial dilution of ATc or DOX. Optical density was recorded after 14 days and normalized to the corresponding strains without drug treatment.

### Animal infection and DOX administration.

All animal studies were performed in biosafety level 2 (BSL2) and BSL3 facilities and approved by the Institutional Animal Care and Use Committee of the New Jersey Medical School, Rutgers University, Newark, NJ. All samples collected from M. tuberculosis-infected animals were handled and processed in the BSL3 facility in compliance with protocols approved by the Institutional Biosafety Committee of the New Jersey Medical School, Rutgers University, Newark, NJ.

For pharmacokinetic studies in uninfected mice, 4- to 6-week-old CD-1 female mice received a modified rodent diet containing 2,000 ppm DOX for the duration indicated in each experiment. Several diet lots from two different manufacturers were tested in pilot experiments. Laboratory rodent diet 5001 with DOX 2,000 ppm (DOX chow) from Purina (catalog number c113-200i) provided the highest and most consistent plasma levels and was selected for all experiments reported here. Blood was collected at various time points for up to 3 days while mice were receiving DOX chow, kept on ice, centrifuged at approximately 2,350 × *g* (5,000 rpm in a table-top centrifuge) for 5 min, and stored at –80°C until analyzed for DOX content.

For plasma and tissue pharmacokinetic studies in infected mice, 8- to 10-week-old female BALB/c mice were infected with a low dose of 100 to 200 CFU M. tuberculosis H37Rv (ATCC 27294) using a full-body inhalation exposure system (Glas-Col) as previously described (42). Mice received DOX chow for 7 days 4 weeks postinfection. For the determination of DOX content in major organs relative to plasma, one group of 3 mice was euthanized on day 7 and organs were collected, weighed, and stored at –80°C until analyzed for DOX content. Blood was collected at the time of euthanasia and processed as described above. To study the time to clearance of DOX from plasma and tissues after DOX chow (received for 7 days) was replaced with a DOX-free diet, groups of 3 mice were euthanized just prior to and then 1, 2, 4, and 7 h and 1, 2, 3, 4, 7, 10, and 14 days after replacement of DOX chow with standard rodent diet. Plasma and lungs were collected, processed, and stored as described above.

For pharmacokinetic studies in rabbits, female New Zealand white (NZW) rabbits (Millbrook Farm, Concord, MA), weighing 2.2 to 2.6 kg, were maintained under specific pathogen-free conditions and fed water and chow *ad libitum*. For dose-finding pharmacokinetic studies in uninfected animals, rabbits received DOX at the following doses/routes: 5 mg/kg intravenously formulated in saline, 20 mg/kg by oral gavage and formulated in deionized water, 24 mg/kg subcutaneously formulated in deionized water, or 15 and 30 mg/kg administered as DOX pills (either half a pill or one full pill of 100 mg doxycycline hyclate; Henry Schein Animal Health, Covetrus, OH) by oral gavage. Rabbit weights were approximately 3.0 to 3.5 kg at the time of dosing.

For plasma and tissue pharmacokinetics in TB-infected NZW rabbits, animals were infected with M. tuberculosis HN878 using a nose-only aerosol exposure system as described (43). At 12 to 20 weeks postinfection, once mature cellular and necrotic lesions had developed, rabbits received LabDiet 5001 supplemented with DOX at 200 ppm, 400 ppm, or 2,000 ppm (Research Diets, Inc., New Brunswick, NJ) for the duration indicated in each experiment.

For pharmacokinetic studies of DOX in a modified diet containing low or no calcium, uninfected rabbits received a gradually phased diet as follows. To test DOX PK in a calcium-free diet, rabbits received 25/75% calcium-free chow mixed with standard high-fiber rabbit chow (Laboratory Rabbit Diet HF 5326; LabDiet)—containing 10,000 ppm or 1% calcium—for 2 days, followed by 50/50% calcium-free chow mixed with standard chow for 2 days, then 75/25% calcium-free chow mixed with standard chow for 2 days, and finally, 100% calcium-free chow for 2 days, at which point they received a single oral dose of 25 mg/kg DOX formulated in deionized water (data shown in Fig. 3B). To test DOX PK in a low-calcium diet (20% of standard calcium content or 2,000 ppm), rabbits received 20/80% calcium-free chow mixed with standard high-fiber rabbit chow for 2 days, followed by 50% calcium-free chow mixed with 50% standard chow for 2 days, and finally, 80/20% calcium-free chow mixed with standard chow for 2 days, at which point DOX was added to the chow at 2,000 ppm final content (data shown in Fig. 3C). Customized calcium-free chow was manufactured with purified ingredients aiming to resemble the dietary parameters of standard high-fiber rabbit chow (18.2% protein, 56.2% carbohydrate, 3.6% fat; per kg of chow: 175 g casein, 390 g corn starch, 25 g maltodextrin, 125 g sucrose, 150 g cellulose, 25 g inulin, 35 g soybean oil, 38.3 g calcium-free mineral mix, 10 g vitamin mix, and 2 g choline bitartrate). Modified rabbit chow was obtained from Research Diets, New Brunswick, NJ.

### *In vitro* pharmacokinetic assays and analytical methods.

Caseum binding and macrophage uptake of DOX were measured as described previously (31, 44).

High-pressure liquid chromatography coupled to tandem mass spectrometry (LC-MS/MS) methods for plasma and whole tissue analysis DOX-HCl and Verapamil were purchased from Sigma-Aldrich. DOX-d3 internal standard was purchased from Toronto Research Chemicals. Drug-free K_2_EDTA plasma and lungs from CD1 mice and NZW rabbits were obtained from BioIVT for use as blank matrices to build standard curves. Neat 1 mg/ml dimethyl sulfoxide (DMSO) stocks DOX were serial diluted in 50/50 acetonitrile (ACN)/Milli-Q water to create neat standards. Control tissue homogenate and study sample homogenate were created by adding 9 parts phosphate-buffered saline (PBS) buffer to 1 part tissue (10× dilution) and shaking the samples using a Fisher bead mill for 1 min at 6,000 × *g* with zirconia beads. Standard, quality control, and study samples for DOX were extracted by adding 10 μl of tissue homogenate or plasma, 10 μl of DOX-d3 internal standard, and 100 μl of extract solvent containing 33% trichloroacetic acid. Extracts were vortexed for 5 min and centrifuged at 2,350 × *g* for 5 min. Then, 100 μl of supernatant was transferred to a 96-well plate for LC-MS/MS analysis. LC-MS/MS analysis was performed on a Sciex Applied Biosystems Qtrap 6500+ triple-quadrupole mass spectrometer coupled to a Shimadzu Nextera X2 ultrahigh-performance liquid chromatography (UHPLC) system to quantify each drug in plasma. Chromatography was performed on an Agilent Zorbax SB-C8 column (2.1 × 30 mm; particle size, 3.5 μm) using a reverse-phase gradient elution with aqueous mobile phase. Milli-Q deionized water with 0.1% formic acid (FA) and 0.1% heptafluorobutyric acid (HFBA) was used for the aqueous mobile phase, and 0.1% FA and 0.1% HFBA in ACN was used for the organic mobile phase. Multiple-reaction monitoring (MRM) of precursor/fragment transitions in electrospray positive-ionization mode was used to quantify the analytes. MRM transitions of 445.20/428.20 and 449.00/432.00 were used for DOX and DOX-d3, respectively. Sample analysis was accepted if the concentrations of the quality control samples were within 20% of the nominal concentration. Data processing was performed using Analyst software version 1.6.2 (Applied Biosystems Sciex).

### Laser-capture microdissection coupled to LC-MS/MS.

Laser-capture microdissection (LCM) sample quantification was carried out according to a previously published protocol (33). Briefly, 1 mg/ml neat DMSO stocks of DOX were serial diluted in 50/50 ACN/Milli-Q water to create neat standards. Control tissue homogenate was created by adding 25.6 parts PBS buffer to 1 part tissue (26.7× dilution) and shaking the samples using a Fisher bead mill for 1 min at 6,000 rpm with zirconia beads. Standard, quality control, and control samples were extracted by adding 2 μl of blank homogenate, 10 μl of neat standard, 5 μl of DOX-d3 internal standard, and 50 μl of extract solvent containing 33% trichloroacetic acid. LCM study samples were extracted that were identical to standards using 2 μl of PBS in place of tissue homogenate. Extracts were bath sonicated for 10 min and centrifuged at 2,350 × *g* for 5 min. Then, 50 μl of supernatant was transferred to a 96-well plate for HPLC-MS/MS analysis. HPLC-MS/MS analysis was performed as described above using the whole-tissue analysis methods.

## Supplementary Material

Supplemental file 1

Supplemental file 2
