# Supplementary material for: Tissue Distribution of Doxycycline in Animal Models of Tuberculosis
Source: Antimicrob Agents Chemother. 2020 Apr 21;64(5):e02479-19. doi: 10.1128/AAC.02479-19 (PMC7179585; doi:10.1128/AAC.02479-19)
Supplement: Supplemental file 1 [file AAC.02479-19-s0001.pdf]

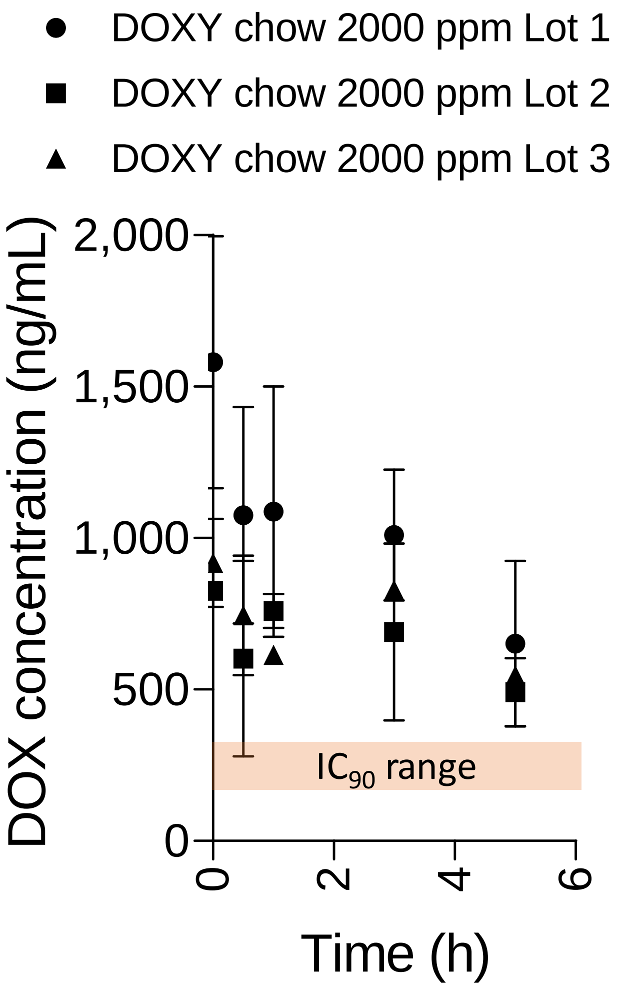

Supplemental Figure 1. Plasma pharmacokinetics of DOX in mice receiving three different batches of 2,000 ppm DOX in standard diet for 7 days. Mean values and standard deviations (error bars) are shown for n = 4 mice per group. The orange shaded window indicates the range of in vitro DOX IC_{90}, or concentration that inhibit 90% of bacterial growth for the three reporter strains shown in Figure 1.

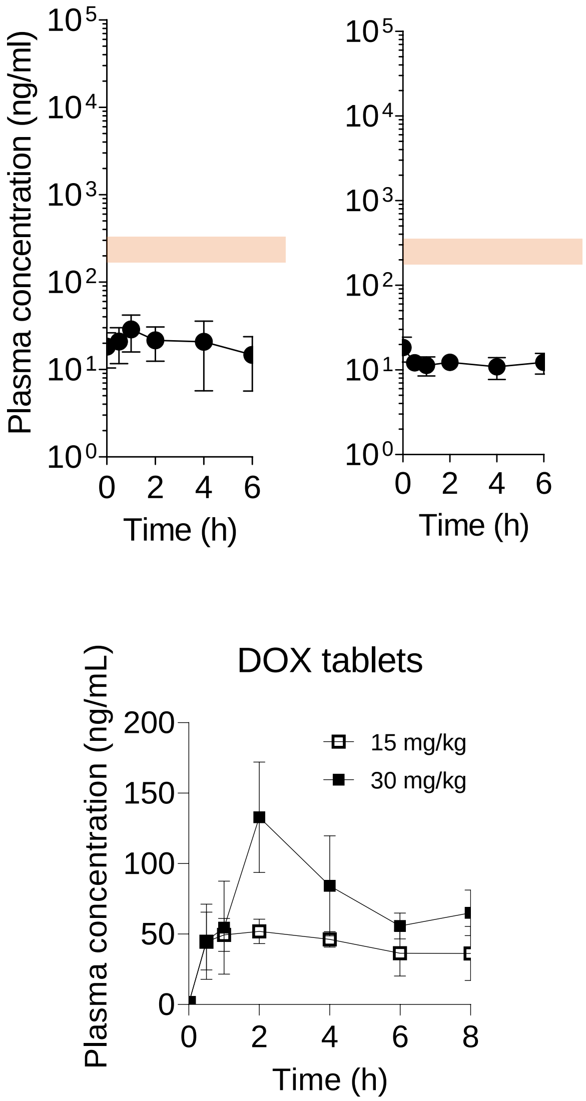

Supplemental Figure 2. **(A)** Plasma pharmacokinetics of DOX in rabbits receiving 200 and 400 ppm DOX in standard chow for 7 days. The orange shaded window indicates the range of in vitro DOX IC_{90}, or concentration that inhibit 90% of bacterial growth for the three reporter strains shown in Figure 1. Mean values and standard deviations (error bars) are shown for n = 3 rabbits per dosing group. **(B)** Plasma pharmacokinetics of DOX in rabbits receiving a single DOX dose in the form of pills at approximately 15 or 30 mg/kg. Mean values and standard deviations (error bars) are shown for n = 4 rabbits per dosing group.

SUPPLEMENTAl TABLE

Table S1. Actual versus target DOX content of 2,000 ppm chow lots

Manufacturer	Lot number	Actual concentration			Target concentration (ng/g)	% difference
		Replicate 1	Replicate 2	Average		
Purina	17041105Hs5.Oi	1620000	1600000	1610000	2000000	-24.2%
Purina	17052302Hs5.Oi	2140000	1850000	1995000	2000000	-0.3%
Purina	17030311HS5.Oi	2210000	1920000	2065000	2000000	3.1%
